# Профессор Д.Я. Шурыгин — основоположник военной эндокринологии (к 100-летию со дня рождения)

**DOI:** 10.14341/probl13308

**Published:** 2023-05-21

**Authors:** В. В. Салухов, С. Б. Шустов

**Affiliations:** Военно-медицинская академия им. С.М. Кирова; Военно-медицинская академия им. С.М. Кирова

**Keywords:** Дорофей Шурыгин, хирургия, эндокринологи

## Abstract

В 2023 г. исполнилось бы 100 лет со дня рождения видного советского эндокринолога, Заслуженного деятеля ­науки РСФСР, главного эндокринолога Министерства обороны СССР (1962–1982 гг.), начальника кафедры терапии №1 для усовершенствования врачей Военно-медицинской академии им. С.М. Кирова (1972–1982 гг.), профессора и генерала-майора медицинской службы Дорофея Яковлевича Шурыгина.

## Abstract (en)

In 2023 it would have been 100 years since the birth of a prominent Soviet endocrinologist, Honored Scientist of the RSFSR, chief endocrinologist of the USSR Ministry of Defense (1962-1982), head of the Department of Therapy No. 1 for the improvement of doctors of the Military Medical Academy. CM. Kirov (1972-1982), Professor and Major General of the Medical Service Dorofei Yakovlevich Shurygin.

В 2023 г. исполнилось бы 100 лет со дня рождения видного советского эндокринолога, Заслуженного деятеля науки РСФСР, главного эндокринолога Министерства обороны СССР (1962–1982 гг.), начальника кафедры терапии №1 для усовершенствования врачей Военно-медицинской академии им. С.М. Кирова (1972–1982 гг.), профессора и генерала-майора медицинской службы Дорофея Яковлевича Шурыгина.

Д.Я. Шурыгин является воплощением особого поколения академической профессуры — фронтовиков, прошедших трудными дорогами Великой отечественной войны, которые, закалившись, впоследствии стяжали славу выдающихся ученых академии и страны, великолепных клиницистов и создателей известнейших научных школ.

Действительно, родившись в небольшом селе русских староверов в Забайкалье, Дорофей, благодаря невероятным жажде знаний и трудолюбию, а также бесспорной одаренности, с отличием окончил десятилетку в Улан-Удэ и поступил в медицинский вуз в далеком Ленинграде. Затем годы военного лихолетья: одновременно с учебой — работа санитаром в уже блокадном городе, эвакуация, подготовка и поступление на военно-медицинский факультет в Москве, блестящее окончание и славный боевой путь военврачом до предместий Берлина. С 1947 г. Д.Я. Шурыгин — адъюнкт при кафедре факультетской терапии Военно-Медицинской академии им. С.М. Кирова, и с этого времени вся последующая его деятельность неразрывно связана с академией. В 1951 г. он успешно защитил кандидатскую диссертацию, посвященную особенностям кроветворения у больных токсическим зобом. Пророческими оказались слова официального оппонента — академика Н.С. Молчанова: «Будем надеяться, что обсуждаемое сейчас исследование Дорофея Яковлевича Шурыгина явится не завершением его научных поисков, а началом новых изысканий, новых достижений». В 1962 г. Д.Я. Шурыгин защитил докторскую диссертацию на тему: «Изменение эндокринной системы при лейкозах и лимфогранулематозе». Пройдя долгий и тернистый путь становления, раскрывший такие незаурядные качества, как твердость характера, умение служить поставленной цели, Д.Я. Шурыгин стал выдающимся ученым и клиницистом, положившим начало многим научным направлениям в Военно-медицинской академии.

В 1969 г. профессор Д.Я. Шурыгин возглавил кафедру факультетской терапии, а в 1972 г. Д.Я. Шурыгин был назначен начальником одной из ведущих клинических кафедр Военно-медицинской академии — кафедры терапии № 1 (для усовершенствования врачей). Его чрезвычайно успешное и продуктивное 10-летнее руководство кафедрой вылилось в развитие клинической и военно-прикладной эндокринологии, а также ассоциированных с этим вопросов кардиологии и терапии. Он разработал новое научное направление — изучение роли эндокринной системы при адаптации организма человека к воздействию экстремальных факторов внешней среды, к условиям военного труда. Основное внимание ученого было сосредоточено на исследовании адаптационно-трофической функции нейроэндокринной системы при травматической, ожоговой, вибрационной болезнях, острой кровопотере, гипокинезии, воздействии химических агентов, под влиянием комплекса факторов высокогорья, высоких широт. Исследования показали, что экстремальные состояния, в которые попадает человеческий организм, вызывают комплексную перестройку гормональной регуляции, сложный каскад изменений функции всех желез внутренней секреции. Благодаря его трудам стало ясно, что эндокринный ответ является очень важной и неотъемлемой характеристикой различных видов боевой терапевтической травмы. В целом под его руководством было выполнено более 200 научных работ, при консультации было защищено 15 докторских и 38 кандидатских диссертаций.

Огромный научный материал, полученный Д.Я. Шурыгиным, его сотрудниками и учениками, позволил оптимизировать консервативную терапию изучаемых состояний, а также обосновать способы коррекции адаптационного процесса и повышения работоспособности человека в экстремальных условиях.

Весьма плодотворной была общественно-научная деятельность профессора Д.Я. Шурыгина. На протяжении 10 лет он был председателем правления Ленинградского научного общества терапевтов им. С.П. Боткина, также являлся членом правления Всесоюзного, Всероссийского научных обществ терапевтов и Всесоюзного научного общества эндокринологов, заместителем председателя ленинградского отделения этого общества. Д.Я. Шурыгин был организатором и участником ряда Всесоюзных и Всероссийских съездов и конференций терапевтов и эндокринологов, а также принимал участие в нескольких международных эндокринологических конгрессах. Последним крупным общественно-научным мероприятием, в организации которого Дорофей Яковлевич принимал самое активное участие, и на котором он выступил с проблемным докладом, был II Всесоюзный съезд эндокринологов, состоявшийся в октябре 1981 года в Ленинграде.

В июле 1982 г. Дорофея Яковлевича не стало. Друзья, коллеги и ученики запомнили Дорофея Яковлевича как человека, который любил искусство, поэзию, музыку, а также сочетал в себе благородство, высокую гражданственность, яркий талант ученого, клинициста и организатора.

Яркий представитель боткинской терапевтической школы, виднейший отечественный эндокринолог, основатель военной эндокринологии профессор Д.Я. Шурыгин оставил обширное научное наследие и воспитал учеников, достойно продолжающих его дело в эндокринологии и терапии. И в этот юбилейный год важно подчеркнуть, что многие научные идеи Д.Я. Шурыгина не потеряли своей актуальности, более того, вызовы настоящего времени настоятельно подтверждают их значение и необходимость дальнейшего развития.

